# Gender differences in quality of life among patients with myasthenia gravis in China

**DOI:** 10.1186/s12955-020-01549-z

**Published:** 2020-09-03

**Authors:** Dong Dong, Marc Ka-chun Chong, Yushan Wu, Henry Kaminski, Gary Cutter, Xianhao Xu, Haifeng Li, Chongbo Zhao, Jian Yin, Siyue Yu, Jianfeng Zhu

**Affiliations:** 1 JC School of Public Health and Primary Care, Faculty of Medicine, The Chinese University of Hong Kong, Hong Kong, SAR China; 2 Department of Neurology, The George Washington University, Washington, DC USA; 3 Department of Biostatistics, University of Alabama at Birmingham, Birmingham, AL USA; 4 Department of Neurology, Beijing Hospital, Beijing, China; 5 Department of Neurology, Xuanwu Hospital, Capital Medical University, Beijing, China; 6 Department of Neurology, Huashan hospital, Fudan University, Shanghai, China; 7 National Center of Gerontology, Institute of Geriatric Medicine, Chinese Academy of Medical Sciences, Beijing, China; 8 School of Social Development and Public Policy, Fudan University, Shanghai, China

**Keywords:** Myasthenia gravis, Health-related quality of life, Gender differences, China

## Abstract

**Background:**

Myasthenia gravis (MG), a chronic neuromuscular disorder, can adversely affect patients’ health-related quality of life (HRQoL), especially in women. The study aimed to evaluate the difference in HRQoL of women and men MG patients and explore the factors that mediate the relationship between gender and HRQoL.

**Methods:**

A cross-sectional study was conducted among 1815 patients with MG in China. The revised 15-item MG quality of life scale (MG-QOL15r) was used to access patients’ HRQoL in overall, physical, social and emotional domains. Socio-demographic information, diagnosis and treatment history, comorbidities, social support, active lifestyle and the MG activities of daily living scale (MG-ADL) were recorded and compared between women and men using the Student’s t-test and Pearson’s Chi-square test. Multivariable regression analyses were conducted to identify independent contributors to HRQoL, especially those affecting different gender.

**Results:**

On average, female patients with MG reported a lower MG-QOL15r score than the males (44.49 ± 29.10 vs 49.32 ± 29.18). The association between gender and patients’ HRQoL interacted with the number of comorbidities across the overall, physical and social domains of patients. As the number of comorbidities increased, the scores of HRQoL decreased and it was faster among females than the males (*p* < 0.05). Moreover, unemployment, exacerbation of the disease, and active lifestyle contributed to the patients’ HRQoL across all domains. Unemployment (β = − 4.99 [95%CI, − 7.80 to − 2.18], *p* < 0.001) and exacerbations (β = − 8.49 [95%CI, − 11.43 to − 5.54], *p* < 0.001) were correlated with poorer HRQoL; while an active lifestyle had a positive impact on HRQoL (β = 0.28 [95%CI, 0.16 to 0.40], *p* < 0.001).

**Conclusions:**

The results indicate that the HRQoL of women MG patients was lower than that of men. The relationship between gender and HRQoL is modulated by the number of comorbidities. Thus, to improve the HRQoL of women MG patients, symptomatic treatments might not be enough, their comorbid conditions should be considered as well. Additionally, employment status, MG exacerbations, and an active lifestyle have been found as determining factors of the patients’ HRQoL, which suggests future interventions should cope with these factors to improve their quality of life.

## Introduction

Myasthenia gravis (MG) is a chronic autoimmune neuromuscular disorder due to autoantibodies formed against the relevant membrane proteins at the neuromuscular junction. The contraction of neuromuscular transmission leads to fluctuating muscular weakness. The symptoms of MG may include weakness of eye muscles (ptosis), difficulty in swallowing and breathing, weakness in the arms, legs, and other muscles [1]. The global incidence of MG is estimated to be 1.7–21.3/1,000,000 person-years and its prevalence is approximately 15–179/1,000,000 [2]. In China, a recent study showed that the estimated annual incidence of MG was 1.55–3.66/100,000 and its estimated prevalence was 2.19–11.07/100,000 in Guangzhou, a provincial capital in South China [3]. But there is no national estimation of either incidence or prevalence in the country.

Although the mortality rate of MG can be reduced dramatically by effective therapies, patients’ health-related quality of life (HRQoL) can still be severely affected by the disease. HRQoL is a multidimensional construct used to measure patients’ health status in physical, mental, emotional and social domains. It also focuses on the impact of disease or treatment on individual well-being [4]. Hence the assessment of patients’ HRQoL has become an increasingly important research area in clinical investigation and health policymaking. When studying the HRQoL of MG patients, the 36-item Short-Form Health Survey (SF-36) has been widely applied to explore the impact of MG on patients’ physical and mental well-beings [5–7]. Studies also revealed that age, occupation and the status of the patients’ thymus were factors significantly influencing the HRQoL of MG patients [8]. More recently, MG-specific quality of life instruments, including the 60-item MG quality of life scale (MG-QOL60) [9], a shortened version of MG-QOL-60, MG-QOL15 [10], and its revised version, MG-QOL15r [11], have become increasingly popular for measuring MG patients’ physical and psychological functions. According to studies where MG specific HRQoL instruments were applied, factors including the severity of disease, dose of prednisone, levels of anxiety and depression could have impact on MG patients’ well-beings as well [12, 13].

Moreover, as an autoimmune disease, MG is found to affect women more than men in terms of prevalence and incidence. The female-to-male ratio of MG patients is generally described as around 2:1; however, it varies with age and/or disease subdivisions [14, 15]. MG occurs more in adult females below 40 years and males above 50 years and that the incidence of generalized MG was significantly higher in women than men [16]. As women are disproportionately affected by MG, their HRQoL might be more compromised than men. A previous study that assessed 2150 patients with MG from German Myasthenia Association using SF-36 showed that physical functioning, vitality, and mental health were found poorer in female patients [17]. These findings were consistent with a study of 1321 MG patients in the United States (US). Measured using MG-QOL15, HRQoL of US female patients with MG was also found to be significantly lower than that of the male patients [18]. Gender, as an important determinant of HRQoL of patients with MG, may also affect the outcomes of treatment. For example, thymectomy has been highlighted as an important factor that could significantly improve the HRQoL of female patients with MG, but not in the males [18]. But the mechanism underneath such gender-related differences in MG is largely unknown. The onset age, disease duration or the efficacy of treatment might interact with gender and mutually impact HRQoL of patients with MG [19].

China might have the largest population of patients with MG in the world. This is the first nationwide study investigating the relationship between gender and HRQoL among Chinese patients with MG. The study aimed to examine differences between men and women patients with respect to HRQoL using the latest version of MG specific HRQoL measure, MG-QOL15r, to explore the factors mediating the relationship between gender and HRQoL.

## Methods

### Study design and subjects

A cross-sectional survey on Chinese patients with MG was conducted online. The online questionnaire was distributed by the Beijing Aili Myasthenia Gravis Care Center (Beijing Aili MG Center) to the patients through a social network platform, SMS (text messaging services), and direct telephone calls between May 2nd, 2018 and February 15th, 2019. Among the 2000 participants enrolled in this study, 43 identified themselves as non-MG patients or not sure about their diagnosis, 93 were under 18 years old when participating the study, and the answers from 49 participants were not valid. Hence, the rest 1815 participants who fit the two inclusion criteria, aged 18 or above and with confirmed diagnosis of MG, were included for data analysis. All participants were required to read the informed consent form presented on the first page of the online survey. If participants agreed to participate in this survey, they clicked the “next page” button at the end of the informed consent form page and were directed to the main body of the questionnaire.

### Data collection

#### Questionnaire

The survey questionnaire was translated and modified from the one used by the US Myasthenia Gravis Patient Registry [19]. The permission for translation and modification was obtained directly from the Registry. Two bilingual English-Chinese speakers translated the questionnaire independently. Then, they discussed the differences and reconciled them until an agreement was achieved. The bilingual version of the questionnaire was then presented to the medical board of Beijing Aili MG Center for comments. Additional questions, such as the use of Traditional Chinese Medicine, questions on social support and active lifestyle, were added to the questionnaire, to tailor to the needs of Chinese patients. The final version of the Chinese MG questionnaire was reviewed and approved by three medical doctors in China who are specialized in MG.

#### Measures

The questionnaire comprised of the following sections: **(1) Socio-demographic** information that includes age, gender, education, employment status, and annual family income. **(2) Diagnosis and treatment history** that includes the age of disease onset, years of diagnosis, disease duration, thymic hyperplasia status, thymic tumor status, thymectomy, history of intensive care unit (ICU), history of MG exacerbation in the past 6 months, and the use of prednisone. **(3) Co-morbidity** was reported by choosing from a list of common chronic diseases and conditions. **(4) The MG activities of daily living scale (MG-ADL)**, which is comprised of eight questions to access disease symptoms and daily activity performance among patients with MG. It was developed based on the quantitative myasthenia gravis score [20]. Among the eight questions, three focused on oropharyngeal functions, two on ocular symptoms, one on respiratory function, and two on upper extremity function. The ratings comprised of a four-point scale ranging from 0 (normal) to 3 (severe). The sum of scores of MG-ADL ranged from 0 to 24, with a higher score indicating a lower functional ability. **(5) Social Support** was measured by the 19-item Medical Outcomes Study Social Support Survey (MOS-SSS), a self-rated survey which contains four subscales including tangible support (e.g. providing financial support or services), positive social interaction (e.g. providing good company), affectionate support (e.g. showing love) and emotional/information support (e.g. giving advice or sharing worries) [21]. Each item has five responses ranging from 1 (none of the time) to 5 (all the time). Scores for each subscale were calculated by averaging the scale items and then were transformed into a value ranging from 0 to 100. **(6) Active lifestyle** was measured with a seven-item scale borrowed from the Chinese General Social Survey [22]. The participants were asked whether they had an active lifestyle in the past year, including, for instance, whether they went out to watch a movie. Each item contains five ratings ranging from 1 (every day) to 5 (never). Items were reversely coded and transformed into a 20 to 100 scale in data analysis. The higher score indicates a more active lifestyle. **(7) MG-QOL15r**, an efficient and user-friendly MG specific HRQoL instrument with fifteen questions, including mobility, MG symptoms, general satisfaction and psychological condition [11]. With each response, there are three options graded from 0 (not at all) to 2 (very much). When analyzing, nine items originally assigned to measure mobility were divided into two sub-scales. Items 4, 6, and 8 were allocated to indicate social quality of life (QoL) and items 5, 7, 10, 12, 13, 15 to physical QoL [13]. The Chinese version of all scales listed above has been validated and shown to have adequate reliability and validity [23–25].

### Statistical analysis

The continuous variables were expressed with means ± standard deviations (SD) or median with interquartile range (25–75%), depending on the parametric or non-parametric distribution of the variables*.* A logarithmic transformation was used to normalize the distribution of variables following a non-normal distribution before using in the parametric analyses. Categorical variables were presented with frequencies and percentages. The Student’s *t*-test and Pearson’s Chi-square test were applied to assess the differences in distributions between women and men for continuous variables and categorical variables, respectively. The internal consistency of MG-QOL15r was examined by Cronbach’s alpha coefficient, and a value greater than 0.70 was considered satisfactory [22]. Multivariable linear regression analyses were used to identify significant factors (age, gender, education, annual household income, disease duration, status of thymectomy, ICU admission, use of prednisone, the number of comorbid conditions, social support, active lifestyle and MG-ADL) associated with each of the three MG-QOL15r dimensions and their interaction terms with gender was examined. Three different models were assessed: (1) socio-demographic factors (except gender), diagnosis and treatment history, the number of comorbid conditions, social support and active lifestyle as well as MG-ADL; (2) Model 1 + Gender, and (3) Model 2 + interaction terms between gender and comorbidities. A *p*-value less than 0.05 was considered statistically significant. All statistical analyses were done on R (version 3.6.1).

## Results

### Socio-demographic factors

As shown in Table 1, out of the 1815 participants included for analysis, 621 (34%) were males, 1194 (66%) were females. Females were significantly younger than males, with the mean age of 39. 82 ± 12.98 years and of males being 43.44 ± 13.59 years (*p* < 0.001). Females were more likely than males to state their employment status as not working (65.83% vs 53.78%, *p* < 0.001). The mean annual household income reported by male patients was higher than that of females (CNY¥ 50,598.95 ± 43,337.15 vs CNY¥ 44,442.01 ± 36,557.39, *p* < 0.001).

**Table 1:** Comparisons of socio-demographics, diagnosis and treatment history, number of comorbid conditions, social support and active lifestyle and MG-ADL by gender

	Men	Valid N	Women	Valid N	*P*-value
Total number	34%	621	66%	1194	
**Socio-demographic variables**					
Average age (Mean ± SD)	43.44 *±* 13.59	621	39.82 *±* 12.98	1194	< 0.001
Educational level		621		1194	< 0.001
Elementary school or lower	45 (7.25%)		141 (11.81%)		
Junior high school	172 (27.70%)		322 (26.97%)		
Senior high school	188 (30.27%)		263 (22.03%)		
Junior college or above	216 (34.78%)		468 (39.20%)		
Employment status		621		1194	< 0.001
Employment	287 (46.22%)		408 (34.17%)		
Unemployment	334 (53.78%)		786 (65.83%)		
Not employed due to MG	238 (71.26%)	238	548 (69.72%)	548	0.658
Annual household income (Mean ± SD)^a^	50,598.95 *±* 43,337.15	621	44,442.01 *±* 36,557.39	1194	< 0.001
**Diagnosis and treatment history**					
Average onset age (Mean ± SD)	34.36 *±* 16.62	621	29.82 *±* 14.87	548	< 0.001
Disease duration (median (range))	4.75 (1.92–9.75)	621	5.54 (2.50–11.40)	548	0.002
Thymic hyperplasia	105 (24.82%)	423	261 (34.66%)	753	< 0.001
Thymic tumor	203 (35.43%)	573	254 (23.01%)	1104	< 0.001
Thymectomy	272 (43.80%)	621	446 (37.35%)	1194	0.009
ICU admission	218 (35.86%)	608	404 (33.47%)	1172	0.597
MG exacerbations	192 (30.92%)	560	421 (35.26%)	1069	=0.05
Prednisone treatment		590		1151	0.989
Current prednisone	295 (50.00%)		572 (49.70%)		
Past prednisone	141 (23.90%)		275 (23.89%)		
Never had prednisone	154 (26.10%)		304 (26.41%)		
**Number of comorbid conditions**		295		570	0.088
One	72 (24.41%)		124 (21.75%)		
Two	37 (12.54%)		56 (9.82%)		
Three or more	41 (13.90%)		60 (10.53%)		
**Social support and active lifestyle**					
Social support (Mean ± SD)		621		1194	
Tangible support	59.77 *±* 28.03		58.61 *±* 27.70		0.398
Affectionate support	43.75 *±* 25.37		47.19 *±* 24.98		< 0.01
Positive social interaction	45.25 *±* 24.78		45.59 *±* 24.92		0.783
Emotional/information support	43.04 *±* 26.39		48.63 *±* 25.56		< 0.001
Active lifestyle	42.42 *±* 12.30	621	40.71 *±* 11.93	1194	< 0.01
**MG-ADL** (Mean ± SD)^**b**^	6.15 *±* 4.54	621	6.75 *±* 4.47	1194	< 0.01

^a^The monetary value was presented in Chinese Yuan (CNY);

^b^The higher score of MG-ADL means worse mobility function

### Diagnosis and treatment history

The average age of disease onset for females was 4.54 years earlier than that of the males (*p* < 0.001). Despite being younger, female patients had a longer disease duration (median = 4.75 years; range 1.92–9.75 years) compared with male patients (median = 5.54 years; range 2.50–11.40 years) (*p* < 0.01). The female group was more likely to have thymic hyperplasia (34.66%) but less likely to report thymic tumor (23.01%) than the male group (24.82 and 35.43%, respectively, *p* < 0.001). More than 43% of the male patients had received a thymectomy operation, the proportion was higher than that of the female patients (37.35%) (*p* < 0.01). Women tended to have a slightly higher rate of MG exacerbations compared with men (35.26% vs 30.92%, *p* = 0.050).

### Comorbid conditions

The detailed information on the prevalence in each comorbid condition among patients with MG is presented in Additional file 1. Autoimmune thyroid disease was more common in female group (15.35%) than in male group (9.62%) (*p* < 0.01). But psoriasis was more common among men (4.62%) than women (2.05%) (*p* < 0.01). There was also a higher prevalence of type 2 diabetes (12.38% vs 5.77%, *p* < 0.001) and high blood pressure in men than women (21.55% vs 11.83%, *p* < 0.001).

### Social support, active lifestyle, and MG-ADL

Among the four domains of social support, both male and female patients had the highest score in tangible support. The mean scores of male patients on the domains, affectionate support, and emotional support, were lower than those by the females and the differences between them were 3.44 (*p* < 0.01) and 5.59 (*p* < 0.001), respectively. On average females reported lower scores on active lifestyle (40.71 ± 11.93) than males (42.42 ± 12.30) (*p* < 0.01). The total score of MG-ADL was equal to or above six in 47.91% of women compared with 41.5% in men. The mean MG-ADL score was higher in women than in men by 0.6 (*p* < 0.01), indicating that female patients with MG might have suffered from more severe MG symptoms than most of the male patients.

### HRQoL measured by MG-QOL15r

The overall internal consistency (Cronbach’s alpha) for MG-QOL15r in our study was excellent (α = 0.953). Cronbach’s alpha for physical QoL was 0.917, for social QoL was 0.877, for emotional QoL was 0.880. The scores achieved for each item of the subscale was firstly reversed and summed to obtain a total subscale score. Then, the score for each subscale was averaged by the maximum subscale value before being multiplied by 100 to yield a value scale ranging from 0 to 100. A higher score indicates a better HRQoL.

The results of MG-QOL15r are summarized in Table 2. Overall, female patients with MG reported poorer HRQoL than male patients (*p* < 0.01). The average MG-QOL15r score for females was 44.49 ± 29.10, whereas that of the males was 49.31 ± 29.18. The overall score of items related to physical QoL in female patients was significantly lower than in male patients by 5.21 (*p* < 0.001); for social QoL, it was lower by 3.5 (*p* < 0.05), and for emotional QoL, it was 5.03 (*p* < 0.01). A figure on individual items of MG-QOL15r compared by gender was provided in Additional file 2.

**Table 2:** Comparison of mean scores of MG-QOL15r and each sub-scale between men and women

	Men	Women	*P*-value
MG-QOL15r	49.32 *±* 29.18	44.49 *±* 29.10	< 0.001
Sub-scales			
Physical QoL	52.22 *±* 29.32	47.01 *±* 29.91	< 0.001
Social QoL	39.43 *±* 33.39	35.93 *±* 32.70	< 0.05
Emotional QoL	50.51 *±* 34.58	45.48 *±* 31.91	< 0.01

Data are presented as mean ± SD

### Multivariable regression analyses

In the multivariable regression analysis, MG-ADL was found to be highly correlated with HRQoL of Chinese patients with MG across all three domains. The worse the MG-ADL, the lower the HRQoL. Whereas the influences from other factors varied (see Tables 3, 4, 5 and 6).

**Table 3:** Results of multivariable regression analyses in overall QoL in MG-QOL15r

Overall QoL	Model 1		Model 2		Model 3	
	β (95% CI)	*P*-value	β (95% CI)	*P*-value	β (95% CI)	*P*-value
**Socio-demographic variables**						
Age	0.13 (0.02 to 0.24)	< 0.05	0.12 (0.01 to 0.23)	< 0.05	0.13 (0.02 to 0.24)	< 0.05
Education	1.19 (− 0.27 to 2.66)	0.111	1.19 (− 0.28 to 2.66)	0.113	1.14 (− 0.33 to 2.61)	0.127
Unemployment	− 4.99 (− 7.80 to − 2.18)	< 0.001	− 4.87 (− 7.70 to − 2.03)	< 0.001	− 4.78 (− 7.61 to − 1.96)	< 0.001
Annual household income	0.00 (0.00 to 0.00)	< 0.01	0.00 (0.00 to 0.00)	< 0.05	0.00 (0.00 to 0.00)	< 0.01
**Diagnosis and treatment history**						
Thymectomy (0 = no; 1 = yes)	− 2.07 (− 4.66 to 0.52)	0.118	− 2.08 (− 4.68 to 0.51)	0.115	− 2.17 (− 4.75 to 0.42)	0.100
MG exacerbations (0 = no; 1 = yes)	− 8.49 (− 11.43 to − 5.54)	< 0.001	− 8.49 (− 11.44 to − 5.54)	< 0.001	− 8.49 (− 11.43 to − 5.55)	< 0.001
Prednisone treatment (0 = never had prednisone)						
Current prednisone	−3.83 (− 6.55 to − 0.21)	< 0.05	− 3.40 (− 6.57 to − 0.23)	< 0.05	− 3.26 (− 6.43 to − 0.10)	< 0.05
Past prednisone	1.33 (− 5.11 to 2.45)	0.489	− 1.32 (− 5.10 to 2.46)	0.493	− 1.22 (− 4.99 to 2.55)	0.526
**Number of comorbid conditions**	0.10 (− 0.63 to 0.82)	0.795	0.08 (− 0.65 to 0.81)	0.826	0.90 (− 0.08 to 1.88)	0.071
**Social support and Active lifestyle**						
Tangible support	−0.03 (− 0.10 to 0.05)	0.458	− 0.03 (− 0.10 to 0.04)	0.432	− 0.03 (− 0.10 to 0.04)	0.446
Affectionate support	0.09 (− 0.03 to 0.22)	0.134	0.10 (− 0.03 to 0.22)	0.121	0.10 (− 0.02 to 0.23)	0.099
Positive social interaction	0.02 (− 0.09 to 0.14)	0.675	0.02 (− 0.09 to 0.13)	0.731	0.02 (− 0.09 to 0.13)	0.739
Emotional/information support	0.00 (−0.09 to 0.10)	0.956	0.01 (−0.09 to 0.10)	0.899	0.00 (−0.10 to 0.10)	0.990
Active lifestyle	0.28 (0.16 to 0.40)	< 0.001	0.28 (0.16 to 0.40)	< 0.001	0.28 (0.16 to 0.40)	< 0.001
**MG-ADL**	− 4.02 (− 4.37 to − 3.67)	< 0.001	− 4.02 (− 4.36 to − 3.67)	< 0.001	− 3.99 (− 4.34 to − 3.64)	< 0.001
**Gender (0 = male; 1 = female)**			− 0.89 (− 3.74 to 1.97)	0.542	0.90 (− 2.29 to 4.09)	0.579
**Gender × Comorbidities**^**a**^					−1.71 (− 3.08 to − 0.34)	< 0.05
**Adjusted R-squared**	0.6155		0.6152		0.6177	

*Abbreviation*: *CI* confidence interval

^a^Number of comorbid conditions

**Table 4:** Results of multivariable regression analyses in physical QoL in MG-QOL15r

Physical QoL	Model 1		Model 2		Model 3	
	β (95% CI)	*P*-value	β (95% CI)	*P*-value	β (95% CI)	*P*-value
**Socio-demographic variables**						
Age	0.04 (− 0.06 to 0.15)	0.419	0.03 (− 0.07 to 0.14)	0.522	0.04 (− 0.07 to 0.15)	0.451
Education	1.12 (− 0.32 to 2.56)	0.127	1.11 (− 0.33 to 2.55)	0.130	1.07 (− 0.37 to 2.50)	0.146
Unemployment	− 4.82 (− 7.58 to − 2.07)	< 0.001	− 4.70 (− 7.48 to − 1.92)	< 0.001	− 4.61 (− 7.38 to − 1.84)	< 0.01
Annual household income	0.00 (0.00 to 0.00)	< 0.05	0.00 (0.00 to 0.00)	< 0.05	0.00 (0.00 to 0.00)	< 0.05
**Diagnosis and treatment history**						
Thymectomy (0 = no; 1 = yes)	−2.70 (− 5.24 to − 0.16)	< 0.05	− 2.72 (− 5.26 to − 0.18)	< 0.05	− 2.81 (− 5.34 to − 0.27)	< 0.05
MG exacerbations (0 = no; 1 = yes)	−7.71 (− 10.60 to − 4.82)	< 0.001	− 7.71 (− 10.60 to − 4.82)	< 0.001	− 7.71 (− 10.59 to − 4.83)	< 0.001
Prednisone treatment (0 = never had prednisone)						
Current prednisone	−3.12 (− 6.22 to − 0.01)	< 0.05	− 3.14 (− 6.25 to − 0.03)	< 0.05	−2.99 (− 6.09 to 0.11)	0.059
Past prednisone	−0.98 (− 4.69 to 2.72)	0.603	− 0.971 (− 4.68 to 2.74)	0.607	−0.86 (− 4.56 to 2.83)	0.646
**Number of comorbid conditions**	0.41 (−0.30 to 1.13)	0.252	0.40 (− 0.31 to 1.11)	0.272	1.25 (0.30 to 2.21)	< 0.05
**Social support and Active lifestyle**						
Tangible support	−0.00 (− 0.07 to 0.07)	0.972	− 0.00 (− 0.07 to 0.07)	0.990	−0.00 (− 0.07 to 0.07)	0.989
Affectionate support	0.06 (−0.07 to 0.18)	0.364	0.06 (−0.06 to 0.18)	0.333	0.07 (−0.06 to 0.19)	0.284
Positive social interaction	−0.03 (− 0.14 to 0.08)	0.609	− 0.03 (− 0.15 to 0.08)	0.559	−0.03 (− 0.15 to 0.08)	0.549
Emotional/information support	0.02 (−0.08 to 0.11)	0.718	0.02 (−0.07 to 0.11)	0.664	0.02 (−0.08 to 0.11)	0.753
Active lifestyle	0.27 (0.16 to 0.39)	< 0.001	0.27 (0.16 to 0.39)	< 0.001	0.27 (0.16 to 0.39)	< 0.001
**MG-ADL**	−4.39 (− 4.73 to − 4.05)	< 0.001	−4.38 (− 4.73 to − 4.04)	< 0.001	−4.36 (− 4.70 to − 4.02)	< 0.001
**Gender (0 = male; 1 = female)**			− 0.93 (− 3.73 to 1.87)	0.516	0.93 (−2.19 to 4.06)	0.557
**Gender × Comorbidities**^**a**^					−1.78 (−3.12 to −0.44)	< 0.01
**Adjusted R-squared**	0.6364		0.6362		0.6389	

*Abbreviation*: *CI* confidence interval

^a^Number of comorbid conditions

**Table 5:** Results of multivariable regression analyses in social QoL in MG-QOL15r

Social QoL	Model 1		Model 2		Model 3	
	β (95% CI)	*P*-Value	β (95% CI)	*P*-Value	β (95% CI)	*P*-Value
**Socio-demographic variables**						
Age	0.21 (0.07 to 0.36)	< 0.01	0.22 (0.07 to 0.37)	< 0.01	0.23 (0.08 to 0.38)	< 0.01
Education	1.17 (− 0.86 to 3.21)	0.258	1.18 (− 0.86 to 3.22)	0.256	1.13 (− 0.91 to 3.16)	0.277
Unemployment	− 5.68 (− 9.57 to − 1.79)	< 0.05	−5.78 (− 9.70 to − 1.85)	< 0.01	−5.68 (− 9.60 to − 1.76)	< 0.01
Annual household income	0.00 (0.00 to 0.00)	0.070	0.00 (0.00 to 0.00)	0.067	0.00 (0.00 to 0.00)	0.058
**Diagnosis and treatment history**						
Thymectomy (0 = no; 1 = yes)	−0.788 (−4.38 to 2.80)	0.667	−0.77 (− 4.37 to 2.82)	0.672	− 0.87 (− 4.46 to 2.72)	0.633
MG exacerbations (0 = no; 1 = yes)	−9.23 (− 13.31 to −5.15)	< 0.001	− 9.23 (− 13.31 to − 5.14)	< 0.001	− 9.22 (− 13.30 to − 5.15)	< 0.001
Prednisone treatment (0 = never had prednisone)						
Current prednisone	−3.79 (− 8.18 to 0.61)	0.091	− 3.77 (− 8.16 to 0.62)	0.093	− 3.61 (− 8.00 to 0.78)	0.107
Past prednisone	−0.13 (− 5.37 to 5.10)	0.960	−0.14 (− 5.38 to 5.10)	0.958	− 0.022 (− 5.25 to 5.21)	0.993
**Number of comorbid conditions**	− 0.31 (− 1.31 to 0.70)	0.550	− 0.29 (− 1.30 to 0.71)	0.566	0.65 (− 0.71 to 2.01)	0.347
**Social support and Active lifestyle**						
Tangible support	− 0.13 (− 0.23 to − 0.03)	< 0.05	− 0.13 (− 0.23 to − 0.03)	< 0.05	−0.13 (− 0.23 to − 0.03)	< 0.05
Affectionate support	0.13 (− 0.04 to 0.30)	0.146	0.12 (− 0.05 to 0.30)	0.159	0.13 (− 0.04 to 0.30)	0.136
Positive social interaction	0.14 (0.02 to 0.30)	0.082	0.14 (0.02 to 0.30)	0.077	0.14 (−0.02 to 0.30)	0.078
Emotional/information support	−0.04 (− 0.17 to 0.10)	0.599	− 0.04 (− 0.17 to 0.09)	0.575	−0.04 (− 0.18 to 0.09)	0.511
Active lifestyle	0.33 (0.16 to 0.49)	< 0.001	0.33 (0.17 to 0.49)	< 0.001	0.33 (0.17 to 0.49)	< 0.001
**MG-ADL**	−3.61 (− 4.09 to − 3.12)	< 0.001	−3.61 (− 4.10 to − 3.13)	< 0.001	−3.58 (− 4.07 to − 3.10)	< 0.001
**Gender (0 = male; 1 = female)**			0.67 (− 3.28 to 4.63)	0.738	2.73 (−1.69 to 7.15)	0.226
**Gender × Comorbidities**^**a**^					−1.97 (−3.87 to −0.07)	< 0.05
**Adjusted R-squared**	0.4509		0.4503		0.4526	

*Abbreviation*: *CI* confidence interval

^a^Number of comorbid conditions

**Table 6:** Results of multivariable regression analyses in emotional QoL in MG-QOL15r

Emotional QoL	Model 1		Model 2		Model 3	
	β (95% CI)	*P*-value	β (95% CI)	*P*-value	β (95% CI)	*P*-value
**Socio-demographic variables**						
Age	0.31 (0.16 to 0.45)	< 0.001	0.29 (0.14 to 0.43)	< 0.001	0.29 (0.14 to 0.44)	< 0.001
Education	1.43 (− 0.56 to 3.43)	0.159	1.41 (− 0.58 to 3.41)	0.164	1.38 (− 0.61 to 3.38)	0.175
Unemployment	−4.79 (− 8.60 to − 0.98)	< 0.05	− 4.47 (− 8.32 to − 0.63)	< 0.05	− 4.41 (− 8.26 to − 0.57)	< 0.05
Annual household income	0.00 (0.00 to 0.00)	< 0.05	0.00 (0.00 to 0.00)	< 0.05	0.00 (0.00 to 0.00)	< 0.05
**Diagnosis and treatment history**						
Thymectomy (0 = no; 1 = yes)	−1.45 (− 4.97 to 2.07)	0.420	− 1.49 (−5.01 to 2.02)	0.405	− 1.56 (− 5.07 to 1.96)	0.386
MG exacerbations (0 = no; 1 = yes)	−10.08 (− 14.08 to − 6.07)	< 0.001	− 10.08 (− 14.08 to − 6.08)	< 0.001	− 10.08 (− 14.08 to − 6.08)	< 0.001
Prednisone treatment (0 = never had prednisone)						
Current prednisone	−3.78 (− 8.09 to 0.52)	0.085	− 3.84 (− 8.14 to 0.47)	0.081	− 3.73 (− 8.04 to 0.57)	0.089
Past prednisone	−3.58 (− 8.71 to 1.55)	0.171	− 3.56 (− 8.69 to 1.58)	0.174	− 3.38 (− 8.61 to 1.65)	0.183
**Number of comorbid conditions**	−0.46 (− 1.44 to 0.53)	0.362	− 0.50 (− 1.48 to 0.49)	0.323	0.10 (−1.23 to 1.43)	0.885
**Social support and Active lifestyle**						
Tangible support	−0.01 (− 0.11 to 0.09)	0.795	− 0.02 (− 0.12 to 0.08)	0.730	−0.02 (− 0.12 to 0.08)	0.740
Affectionate support	0.18 (0.01 to 0.35)	< 0.05	0.19 (0.02 to 0.36)	< 0.05	0.19 (0.02 to 0.36)	< 0.05
Positive social interaction	0.07 (−0.09 to 0.22)	0.384	0.06 (−0.10 to 0.21)	0.467	0.06 (−0.10 to 0.21)	0.471
Emotional/information support	0.00 (−0.13 to 0.13)	0.966	0.01 (−0.12 to 0.14)	0.923	0.00 (−0.13 to 0.13)	0.972
Active lifestyle	0.25 (0.09 to 0.41)	< 0.01	0.25 (0.09 to 0.40)	< 0.01	0.25 (0.09 to 0.41)	< 0.01
**MG-ADL**	−3.32 (−3.80 to −2.85)	< 0.001	− 3.31 (− 3.79 to − 2.84)	< 0.001	−3.29 (− 3.77 to − 2.82)	< 0.001
**Gender (0 = male; 1 = female)**			− 2.33 (− 6.20 to 1.55)	0.238	−1.03 (− 5.37 to 3.30)	0.640
**Gender × Comorbidities**^**a**^					−1.24 (−3.10 to 0.63)	0.192
**Adjusted R-squared**	0.4497		0.45		0.4505	

*Abbreviation*: *CI* confidence interval

^a^Number of comorbid conditions

### Overall quality of life

**Fig. 1:**
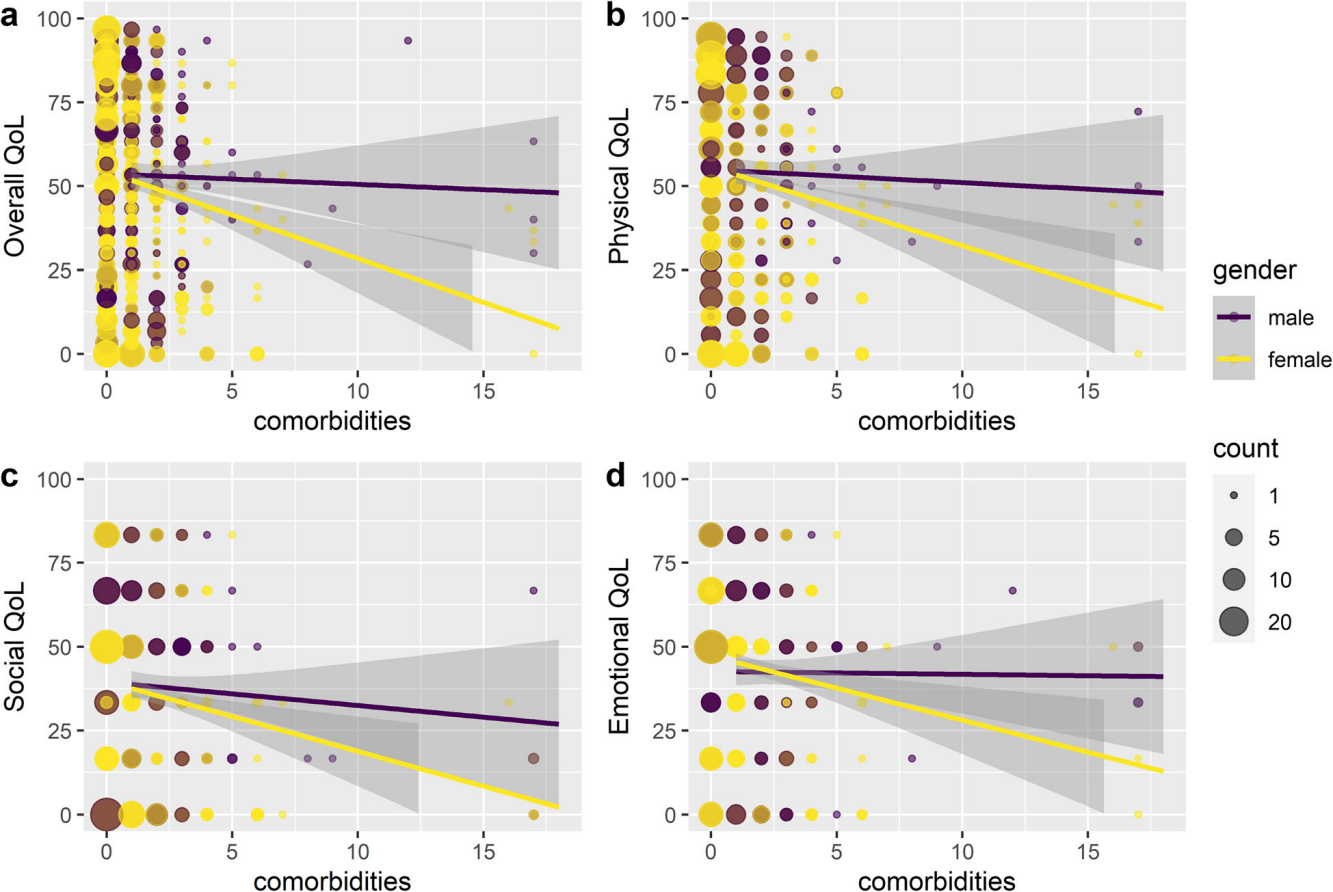
Gender difference in the relationship between number of comorbidities, overall QoL, and each domain in QoL

For the overall quality of life, age, employment status, annual household income, the occurrence of MG exacerbations, current use of prednisone and active lifestyle were significantly associated with HRQoL of patients with MG (Table 3). The results from Model 1 showed unemployment was highly correlated with the poorer overall QoL (β = − 4.99 [95%CI, − 7.80 to − 2.18], *p* < 0.001). The experience of MG exacerbations in the past 6 months was also highly correlated with poorer overall QoL (β = − 8.49 [95%CI, − 11.43 to − 5.54], *p* < 0.001). In Model 2, no significant main effect of gender was found. But in Model 3, there was a statistically significant interaction between gender and the number of comorbidities, indicating that as the number of comorbidities increased, the HRQoL decreased faster in the female group than in male group (β = − 1.71 [95%CI, − 3.08 to − 0.34], *p* < 0.05; see Fig. 1a).

### Physical quality of life

The participants who reported as unemployed, having undergone thymectomy, experienced MG exacerbations and currently being treated with prednisone tended to have a poorer physical QoL (Table 4). Higher annual household income and active lifestyle were positively associated with higher physical QoL. No statistically significant association between social support and physical QoL was found. As with overall QoL, the result in the Model 2 showed gender had no significant impact on physical QoL. Similarly, a statistically significant interaction between gender and the number of comorbidities was found in the Model 3, suggesting that females with more comorbid conditions might experience poorer physical QoL than the males (β = − 1.78 [95%CI, − 3.12 to − 0.44], *p* < 0.01; see Fig. 1b).

### Social quality of life

Older age and more active lifestyle linked to higher scores in items related to the social domain in MG-QOL15r (Table 5). Unemployment, MG exacerbations, and tangible social support negatively impacted on social QoL. Neither thymectomy nor the use of prednisone has no significant influence on the social QoL. The results in the Model 2 suggested gender has no main effect on social QoL, whereas in the Model 3, the gender by comorbidities interaction was significant (β = − 1.97 [95%CI, − 3.87 to − 0.07], *p* < 0.05; see Fig. 1c). This also means social QoL of female patients with MG was more negatively affected by the number of comorbidities.

### Emotional quality of life

All socio-demographic variables except education were associated with emotional QoL in the first step (Table 6). The poor emotional QoL was related to MG exacerbations. Affectionate support and active lifestyle impacted positively on higher emotional QoL. No significant effect was found by gender or the gender by comorbidity interaction on emotional QoL in the Model 3.

## Discussion

The objective of this study was to assess the impact of gender on the HRQoL of patients with MG. The study showed that Chinese female patients with MG consistently scored less than males on physical, social, and emotional domains of HRQoL. However, according to the results of multivariable regression analyses, gender has no main effect on the quality of life across the three domains with the full adjustment of other variables included in the models. In this study, we identified that the number of comorbidities interacted with gender and could modulate the relationship between gender and HRQoL for patients with MG in China.

Consistent with previous studies [18], we found that female patients with MG exhibited a higher degree of disability compared to males, as evidenced by a higher MG-ADL score (or worse function) reported by the females. There might be several possible explanations for the gender differences in disability. First, MG might be more severe in females than in males, possibly because MG symptoms can be affected by menses, pregnancy and postpartum changes in hormone [26, 27] Second, sex-related differences in drug pharmacokinetics and pharmacodynamics may be attributed to differences in body composition and physiology, which may lead to the differential effects of treatment in males and females with MG. Studies have revealed that patients with refractory MG – that is, MG patients do not respond well or show intolerance to therapy – are typically to be females [28–30]. Third, female MG patients might be subject to insufficient treatment with the relatively low dosage of drugs. A previous study on gender differences in prednisone adverse effects showed that compared to males, female MG patients are less willing to accept a dose increase due to the concern about potential adverse effect [19]. Having intolerable adverse effects may contribute to the medication noncompliance of female MG patients, which could cause MG to worsen.

In addition, the gender difference in disability can also be resulted from their differences in receiving thymectomy. A randomized trial of thymectomy in MG has proved that for MG patients, their physical capabilities can be significantly improved by the surgery [31]. However, our study finds that fewer female patients had received thymectomy than the males (37.35% vs 43.80%, *p* < 0.01), which may in turn explain why the female MG patients in China has worse MG-ADL.

Like many previous studies [32], our study also found a strong correlation between MG-ADL and MG-QOL. It is therefore not surprising to see that female patients with MG exhibited poorer HRQoL than the male patients. This finding is consistent with findings from the US that also showed a lower HRQoL among female patients than the males [18]. Although need to be further explored, such a gender difference among MG patients might be explained as follows.

Patients with MG have a higher risk of developing one or more autoimmune disorders than the general population [33, 34]. Sex differences in the prevalence of other autoimmune disorders in MG have been reported [34]. Consistent with previous studies [33], our study reveals that a significantly higher proportion of females are affected by autoimmune thyroid disease, while the prevalence of psoriasis is higher among males. Among non-autoimmune comorbidities, type 2 diabetes and high blood pressure are more commonly seen in male patients with MG, which might be associated with older age [18]. Both autoimmune and non-autoimmune comorbidities are suspected to impair patients’ physical and mental functions of patients, thus reducing their self-care ability. As indicated by Usha K. Misra1 et al., the MG patients with more than two concomitant comorbidities are associated with poor outcome [35]. However, the role of comorbidities of HRQoL between men and women with MG remains unknown in the literature.

In the present study, we showed that, with an increasing burden of comorbidities, the negative impact on physical and social components of HRQoL is stronger in the female group than in their male counterparts adjusted for other variables. Concomitant disorders are more likely to develop in the early-onset of MG rather than in late-onset MG [34–36]. Due to the earlier onset of concordant disorders, female patients with MG may endure adverse impact of coexisting disorders longer than males of the same age, which could result in poorer HRQoL for females. Another possible explanation is that patients of different gender may perceive the adverse effects of medical treatments differently. Prior studies reported that the adverse effects of prednisone were related to changes in appearance such as round face and weight gain which might be more likely to be negative affect for female patients with MG than males [19]. It can be further assumed that women with multiple comorbidities tend to experience poorer HRQoL than men due to the gender differences in adverse effects of therapies for these comorbidities as well. Yet, it is unclear why the HRQoL of female patients with MG depends on the number of comorbidities more than that of the male patients. Future research is urgently needed to explore this area of study.

By and large, most findings from this study are consistent with previous studies in countries other than China. From the socio-demographic perspective, our data suggest that younger age is associated with poorer overall, social and emotional QoL. This result is similar to a prior study showing younger patients with MG report more symptoms and lower HRQoL [37]. Unemployment is found to be significantly associated with worse HRQoL across all domains and most strongly related to the social domain. This is not surprising. Previous studies showed that employment is often correlated with higher HRQoL [17]. Compared with the unemployed group, employed individuals are more satisfied with extrinsic values (e.g., higher income, career advancement, disability compensation) and intrinsic values (e.g., sense of accomplishment [38]. Those who are employed also have more opportunities to form positive social interactions and thus to have a better social QoL.

From the medical perspective, MG exacerbation is found to have negative impacts on HRQoL across the three domains in both genders. Kulkantrakorn and others demonstrate that reduced HRQoL was mainly determined by the frequency of MG symptoms [39]. Boldingh and colleagues also showed that remission and absence of generalized symptoms are important factors for better HRQoL among patients with MG [40]. Our findings provided further evidence to support these conclusions. Moreover, similar to other studies in different countries (e.g., Cutter et al., 2019 in the US [37], Cioncoloni et al., 2016 in Italy [41]), abilities of Chinese patients with MG to perform daily activities (measured by MG-ADL) also had positive impact on their HRQoL. Although prednisone has been proved as an effective therapeutic treatment in patients with MG [42], it was also found that its adverse effects in both long-term and short-term can negatively impact on HRQoL [13] and that total dose of oral prednisolone during the last 1 year had a significant negative effects on patients’ HRQoL [43] In our study, reduced overall and physical HRQoL are more closely associated with patients who were under prednisone treatment during the time of survey. However, such findings should be interpreted with cautions since they only show association, rather than causality, between the current use prednisone and HRQoL. Moreover, since the actual dose of prednisolone taken by the patients was not available, future study needs to further explore the association between the dosage of oral steroids and MG patients’ HRQoL.

From the sociological point of view, few studies explored the role of social support and active lifestyle in the HRQoL of people with MG. In our study, of the four dimensions of social support, only affectionate support is positively associated with emotional QoL but interestingly not with other domains. Tangible support, considered to be the most common support to benefit physical and psychological outcomes, had no significant influence on physical or emotional QoL of the patients. Prior studies demonstrated inadequate tangible support is a risk factor for death and functional deterioration [44]. However, since most participants enrolled in our study had moderate impairment to their physical capabilities or did not have very active MG symptoms, tangible support would not have been indispensable in their lives and its impact on their HRQoL is not as significant as on who did not participate in our survey. Moreover, we did observe that tangible support is inversely correlated with higher social QoL. This may imply that too much tangible support or the need for it may hamper patients’ willingness to be socially active or, at least, it may defeat the necessities to go out and interact with others. However, further study is needed to explore such an inverse correlation.

Regarding the active lifestyle, it is well-documented that having an active lifestyle correlates with a better HRQoL [45]. This is consistent with the present result that active lifestyle links to better HRQoL across the three domains. Enjoyment in an active lifestyle provides the opportunities of doing physical exercises, establishing and maintaining social relationships [46]. Moreover, involvement in meaningful activities contributes to skill development and life satisfaction, keeping in mind that being able to have an active lifestyle may self-select those in whom the disease is milder or under better control.

These socio-demographic, medical, and sociological factors are important as they manifest significant differences between female and male patients with MG in relation to other aspects of their conditions. The Chinese female patients with MG are found to be younger, with the higher unemployment rate, a higher proportion of MG exacerbations, less active lifestyle, and worse MG-ADL, all of which can be regarded as providing further evidence to the reason behind lower HRQoL of the female patients than of the males. Even if the females have higher affectionate and emotional/information social support, neither are found to be related to patients’ HRQoL. But future research is critical to further disentangle the relationship between gender and HRQoL among MG patients.

### Limitations

There are several limitations to our study. First, although the participants were recruited via a national MG patient organization and members of the organization must present a valid proof of diagnosis when register, the possibility that some participants were not real MG patients cannot be fully ruled out. Second, since the questionnaires were self-reported, more accurate assessment of the patients’ clinical status was not available. Moreover, recall bias may exist in questions related to MG disease history such as onset age, disease duration and so on. Third, due to the voluntary nature, selection bias may occur in the current study. Patients who volunteered to participate in the survey might be healthier, more educated and more mentally intact, and thus may not constitute an entirely representative sample of all the patients with MG in China. Fourth, the dose of prednisone may affect the HRQoL of patients with MG, which was not include in the present study. Future study is recommended to further explore the effect of the dose of prednisone in Chinese patients with MG. At last, there are findings inconsistent with previous studies that deserve more in-depth investigations. For example, according to the US MG patient registry, the proportion of females having thymic tumor is significantly higher than that of the males. However, our study shows the opposite. Whether or not such discrepancy is caused by the study sample or by race is worth to be further studied.

## Conclusions

In conclusion, this study reveals that, in China, the HRQoL of female patients with MG is significantly lower than that of the males. However, the influence of gender on HRQoL is not direct, rather it is modulated by the number of comorbidities. Given the females had a much higher comorbidity burden than males, management on comorbid conditions is of great important in minimizing the gender differences in HRQoL between Chinese women and men with MG. In addition, employment status, MG exacerbations, and an active lifestyle have been found as determining factors of the patients’ HRQoL, which suggests that future interventions should cope with these factors to improve their HRQoL.

## Supplementary information

**Additional file 1: Table S1.** Comparisons of comorbid conditions by gender.

**Additional file 2: Figure S1.** Mean values of individual item in MG-QOL15r compared by gender.

## Data Availability

The data that support the findings of the current study are available from the corresponding authors on reasonable request.

## Abbreviations

MG: Myasthenia gravis
HRQoL: Health-related quality of life
QoL: Quality of life
MG-ADL: The MG activities of daily living scale
MG-QOL15r: The revised 15-item MG quality of life scale
SF-36: The 36-item Short-Form Health Survey
ICU: Intensive care unit

## References

[1] Gilhus NE (2016). Myasthenia gravis. N Engl J Med.

[2] Carr AS, Cardwell CR, McCarron PO, McConville J (2010). A systematic review of population based epidemiological studies in myasthenia gravis. BMC Neurol.

[3] Fang W, Li Y, Mo R, Wang J, Qiu L, Ou C (2020). Hospital and healthcare insurance system record–based epidemiological study of myasthenia gravis in southern and northern China. Neurol Sci.

[4] Karimi M, Brazier J (2016). Health, health-related quality of life, and quality of life: what is the difference?. Pharmacoeconomics..

[5] Basta IZ, Pekmezović TD, Perić SZ, Kisić-Tepavčević DB, Rakočević-Stojanović VM, Stević ZD, Lavrnić DV (2012). Assessment of health-related quality of life in patients with myasthenia gravis in Belgrade (Serbia). Neurol Sci.

[6] Kulkantrakorn K, Jarungkiatkul W (2010). Quality of life of myasthenia gravis patients. J Med Assoc Thail.

[7] Padua L, Evoli A, Aprile I, Caliandro P, Mazza S, Padua R, Tonali P (2001). Health-related quality of life in patients with myasthenia gravis and the relationship between patient-oriented assessment and conventional measurements. Neurol Sci.

[8] Yang Y, Zhang M, Guo J, Ma S, Fan L, Wang X (2016). Quality of life in 188 patients with myasthenia gravis in China. Int J Neurosci.

[9] Mullins LL, Carpentier MY, Paul RH, Sanders DB (2008). Disease-specific measure of quality of life for myasthenia gravis. Muscle Nerve.

[10] Burns TM, Conaway MR, Cutter GR, Sanders DB (2008). Less is more, or almost as much: a 15-item quality-of-life instrument for myasthenia gravis. Muscle Nerve.

[11] Burns TM, Sadjadi R, Utsugisawa K, Gwathmey KG, Joshi A, Jones S (2016). International clinimetric evaluation of the MG-QOL15, resulting in slight revision and subsequent validation of the MG-QOL15r. Muscle Nerve.

[12] Mourão AM, Gomez RS, Barbosa LSM, Freitas DS, Comini-Frota ER, Kummer A (2016). Determinants of quality of life in Brazilian patients with myasthenia gravis. Clinics (Sao Paulo).

[13] Utsugisawa K, Suzuki S, Nagane Y, Masuda M, Murai H, Imai T (2014). Health-related quality-of-life and treatment targets in myasthenia gravis. Muscle Nerve.

[14] Jacobson DL, Gange SJ, Rose NR, Graham NMH (1997). Epidemiology and estimated population burden of selected autoimmune diseases in the United States. Clin Immunol Immunopathol.

[15] Yu YL, Hawkins BR, Ip MSM, Wong V, Woo E (1992). Myasthenia gravis in Hong Kong Chinese. Acta Neurol Scand.

[16] Grob D, Brunner N, Namba T, Pagala M (2008). Lifetime course of myasthenia gravis. Muscle Nerve.

[17] Twork S, Wiesmeth S, Klewer J, Pöhlau D, Kugler J (2010). Quality of life and life circumstances in German myasthenia gravis patients. Health Qual Life Outcomes.

[18] Lee I, Kaminski HJ, Xin H, Cutter G. Gender and quality of life in myasthenia gravis patients from the myasthenia gravis foundation of America registry. Muscle Nerve. 2018. 10.1002/mus.26104. 10.1002/mus.26104 29466829

[19] Lee I, Kaminski HJ, McPherson T, Feese M, Cutter G (2018). Gender differences in prednisone adverse effects. Neurol Neuroimmunol Neuroinflamm.

[20] Barohn RJ, McIntire D, Herbelin L, Wolfe GI, Nations S, Bryan WW (1998). Reliability testing of the quantitative myasthenia gravis score. Ann N Y Acad Sci.

[21] Sherbourne CD, Stewart AL (1991). The MOS social support survey. Soc Sci Med.

[22] Bian Y, Li L (2012). The Chinese general social survey (2003-8). Chin Sociological Rev.

[23] Yu DSF, Lee DTF, Woo J (2004). Psychometric testing of the Chinese version of the medical outcomes study social support survey (MOS-SSS-C). Res Nurs Health.

[24] Liu P, Li T (2017). Application of myasthenia gravis-quality of Life-15 in Chinese patients with myasthenia gravis. Stroke Neurological Dis.

[25] Gao X (2013). Evaluation of the scales measuring severity of myasthenia gravis.

[26] Boldingh MI, Maniaol AH, Brunborg C, Weedon-Fekjær H, Verschuuren JJGM, Tallaksen CME (2016). Increased risk for clinical onset of myasthenia gravis during the postpartum period. Neurology..

[27] Leker RR, Karni A, Abramsky O (1998). Exacerbation of myasthenia gravis during the menstrual period. J Neurol Sci.

[28] Schneider-Gold C, Hagenacker T, Melzer N, Ruck T (2019). Understanding the burden of refractory myasthenia gravis. Ther Adv Neurol Disord.

[29] Sudulagunta SR, Sepehrar M, Sodalagunta MB, Settikere Nataraju A, Bangalore Raja SK, Sathyanarayana D (2016). Refractory myasthenia gravis - clinical profile, comorbidities and response to rituximab. Ger Med Sci.

[30] Suh J, Goldstein JM, Nowak RJ (2013). Clinical characteristics of refractory myasthenia gravis patients. Yale J Biol Med.

[31] Wolfe GI, Kaminski HJ, Sonnett JR, Aban IB, Kuo H-C, Cutter GR (2016). Randomized trial of thymectomy in myasthenia gravis. J Thorac Dis.

[32] Muppidi S, Wolfe GI, Conaway M, Burns TM (2011). MG-ADL: still a relevant outcome measure. Muscle Nerve.

[33] Sardu C, Cocco E, Mereu A, Massa R, Cuccu A, Marrosu MG (2012). Population Based Study of 12 Autoimmune Diseases in Sardinia, Italy: Prevalence and Comorbidity. PLoS One.

[34] Nacu A, Andersen JB, Lisnic V, Owe JF, Gilhus NE (2015). Complicating autoimmune diseases in myasthenia gravis: a review. Autoimmunity..

[35] Misra UK, Kalita J, Singh VK, Kumar S (2020). A study of comorbidities in myasthenia gravis. Acta Neurol Belg.

[36] Christensen PB, Jensen TS, Tsiropoulos I, Sørensen T, Kjaer M, Højer-Pedersen E (1995). Associated autoimmune diseases in myasthenia gravis. A population-based study. Acta Neurol Scand.

[37] Cutter G, Xin H, Aban I, Burns TM, Allman PH, Farzaneh-Far R (2019). Cross-sectional analysis of the myasthenia gravis patient registry: disability and treatment. Muscle Nerve.

[38] Campbell A, Converse PE, Rodgers WL (1976). The quality of American life: perceptions, evaluations, and satisfactions.

[39] Kulkantrakorn K, Sawanyawisuth K, Tiamkao S (2010). Factors correlating quality of life in patients with myasthenia gravis. Neurol Sci.

[40] Boldingh MI, Dekker L, Maniaol AH, Brunborg C, Lipka AF, Niks EH (2015). An up-date on health-related quality of life in myasthenia gravis -results from population based cohorts. Health Qual Life Outcomes.

[41] Cioncoloni D, Casali S, Ginanneschi F, Carone M, Veronica B, Rossi A, Giannini F (2016). Major motor-functional determinants associated with poor self-reported health-related quality of life in myasthenia gravis patients. Neurol Sci.

[42] Evoli A, Batocchi AP, Palmisani MT, Lo Monaco M, Tonali P (2004). Long-term results of corticosteroid therapy in patients with myasthenia gravis. Eur Neurol.

[43] Nagane Y (2013). Clinical factors affecting quality of life and treatment targets in patients with myasthenia gravis. Rinsho Shinkeigaku.

[44] Woloshin S, Schwartz LM, Tosteson AN, Chang CH, Wright B, Plohman J, Fisher ES (1997). Perceived adequacy of tangible social support and health outcomes in patients with coronary artery disease. J Gen Intern Med.

[45] Giorgino KB, Blalock SJ, DeVellis RF, DeVellis BM, Keefe FJ, Jordan JM (1994). Appraisal of and coping with arthritis-related problems in household activities, leisure activities, and pain management. Arthritis Care Res.

[46] Brajša-Žganec A, Merkaš M, Šverko I (2011). Quality of life and leisure activities: how do leisure activities contribute to subjective well-being?. Soc Indic Res.

