# Supplementary material for: Gender differences in quality of life among patients with myasthenia gravis in China
Source: Health Qual Life Outcomes. 2020 Sep 3;18:296. doi: 10.1186/s12955-020-01549-z (PMC7470440; doi:10.1186/s12955-020-01549-z)
Supplement: Supplementary file 1 — Additional file 1: Table S1. Comparisons of comorbid conditions by gender. [file 12955_2020_1549_MOESM1_ESM.docx]

Supplementary Table 1. Comparisons of comorbid conditions by gender

|  | Men | Women | P-value |
| --- | --- | --- | --- |
| Type 1 diabetes | 28/525 (5.33%) | 45/1034 (4.35%) | 0.459 |
| Rheumatoid arthritis | 25/515 (4.85%) | 53/1001 (5.29%) | 0.807 |
| Autoimmune thyroid | 48/499 (9.62%) | 150/977 (15.35%) | <0.01 |
| Systemic lupus erythematosus | 14/533 (2.63%) | 22/1066 (2.06%) | 0.592 |
| Multiple sclerosis | 10/525 (1.90%) | 16/1052 (1.52%) | 0.723 |
| Psoriasis | 25/541 (4.62%) | 22/1072 (2.05%) | <0.01 |
| Inflammatory bowel disease | 22/497 (4.43%) | 26/1030 (2.52%) | 0.066 |
| B12 deficiency | 25/434 (5.76%) | 62/835 (7.43%) | 0.319 |
| Neuropathy | 25/488 (5.12%) | 34/962 (3.53%) | 0.192 |
| Asthma | 22/523 (4.21%) | 39/1052 (3.71%) | 0.730 |
| COPD | 11/516 (1.77%) | 16/1029 (1.55%) | 0.542 |
| Type 2 diabetes | 66/533 (12.38%) | 61/1058 (5.77%) | <0.001 |
| High blood pressure | 117/543 (21.55%) | 126/1065 (11.83%) | <0.001 |
| Osteoporosis | 82/477 (17.19%) | 180/921 (19.54%) | 0.319 |
| High cholesterol | 92/484 (19.01%) | 156/985 (15.84%) | 0.147 |
| Depression | 47/464 (10.13%) | 97/913 (10.62%) | 0.849 |
| Anxiety | 58/457 (12.69%) | 112/894 (12.53%) | 1 |
| Cancer | 21/574 (3.66%) | 33/1136 (2.90%) | 0.487 |

Abbreviation: COPD, chronic obstructive pulmonary disease.
