# Supplementary material for: Gender differences in quality of life among patients with myasthenia gravis in China
Source: Health Qual Life Outcomes. 2020 Sep 3;18:296. doi: 10.1186/s12955-020-01549-z (PMC7470440; doi:10.1186/s12955-020-01549-z)
Supplement: Supplementary file 2 — Additional file 2: Figure S1. Mean values of individual item in MG-QOL15r compared by gender. [file 12955_2020_1549_MOESM2_ESM.docx]

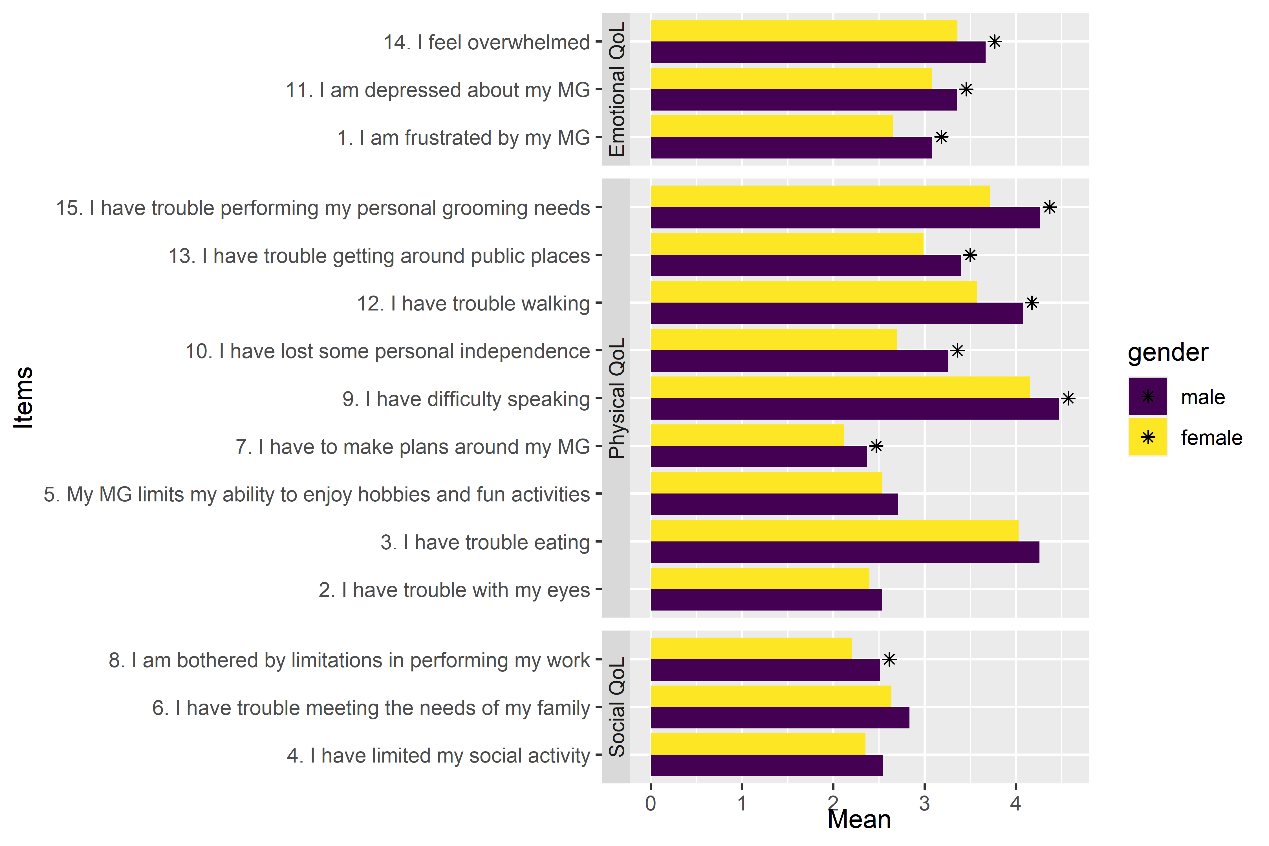


Supplementary Figure 1: Mean values of individual item in MG-QOL15r compared by gender

Note. * indicates the difference between male and female patients was statistically significant.
